# Novel opportunities from bioimaging to understand the trafficking and maturation of intracellular pulmonary surfactant and its role in lung diseases

**DOI:** 10.3389/fimmu.2023.1250350

**Published:** 2023-08-10

**Authors:** María José Garcia, Luciano Amarelle, Leonel Malacrida, Arturo Briva

**Affiliations:** ^1^ Unidad Academica de Fisiopatología, Hospital de Clínicas, Facultad de Medicina, Universidad de la República, Montevideo, Uruguay; ^2^ Advanced Bioimaging Unit, Institut Pasteur de Montevideo & Universidad de la República, Montevideo, Uruguay; ^3^ Unidad Academica de Medicina Intensiva, Hospital de Clínicas, Facultad de Medicina, Universidad de la República, Montevideo, Uruguay

**Keywords:** pulmonary surfactant, lamellar bodies, fluorescence, microscopy, hyperspectral imaging, phasor plot

## Abstract

Pulmonary surfactant (PS), a complex mixture of lipids and proteins, is essential for maintaining proper lung function. It reduces surface tension in the alveoli, preventing collapse during expiration and facilitating re-expansion during inspiration. Additionally, PS has crucial roles in the respiratory system’s innate defense and immune regulation. Dysfunction of PS contributes to various respiratory diseases, including neonatal respiratory distress syndrome (NRDS), adult respiratory distress syndrome (ARDS), COVID-19-associated ARDS, and ventilator-induced lung injury (VILI), among others. Furthermore, PS alterations play a significant role in chronic lung diseases such as chronic obstructive pulmonary disease (COPD) and idiopathic pulmonary fibrosis (IPF). The intracellular stage involves storing and releasing a specialized subcellular organelle known as lamellar bodies (LB). The maturation of these organelles requires coordinated signaling to organize their intracellular organization in time and space. LB’s intracellular maturation involves the lipid composition and critical processing of surfactant proteins to achieve proper functionality. Over a decade ago, the supramolecular organization of lamellar bodies was studied using electron microscopy. In recent years, novel bioimaging tools combining spectroscopy and microscopy have been utilized to investigate the *in cellulo* intracellular organization of lamellar bodies temporally and spatially. This short review provides an up-to-date understanding of intracellular LBs. Hyperspectral imaging and phasor analysis have allowed identifying specific transitions in LB’s hydration, providing insights into their membrane dynamics and structure. A discussion and overview of the latest approaches that have contributed to a new comprehension of the trafficking and structure of lamellar bodies is presented.

## 1 Biogenesis and intracellular surfactant trafficking

Pulmonary surfactant (PS) is a complex mixture of lipids and proteins that covers the epithelial lining fluid at the surface of the alveoli. It is synthesized and secreted by alveolar type II cells (ATII), primarily consisting of phospholipids, cholesterol, and four specific proteins. The lipid fraction constitutes approximately 90% of the total mass, with phospholipids accounting for 80% and dipalmitoylphosphatidylcholine (DPPC). This phospholipid is the most abundant lipid in the PS and is primarily responsible for its tensoactive properties (1). This lipid structure reduces surface tension at the air-liquid interface in the alveoli, preventing alveolar collapse at the end of expiration and facilitating PS re-expansion during inspiration. PS components are synthesized in ATII cells as lamellar bodies (LB). At the air-liquid interphase, it forms a lipid monolayer, with phospholipids playing a pivotal role in surface tension reduction. PS is recycled as vesicles being reabsorbed by ATII cells and macrophages (2, 3). In addition to its biophysical properties, PS plays a crucial role in the innate defense of the respiratory system. Hydrophilic proteins SP-A and SP-D, known as collectins, can bind microorganisms and regulate immune cell activation, while hydrophobic proteins SP-B and SP-C interact with lipids and are essential for the assembly and transfer of PS to the alveolar surface (1, 4).

**Figure 1:**
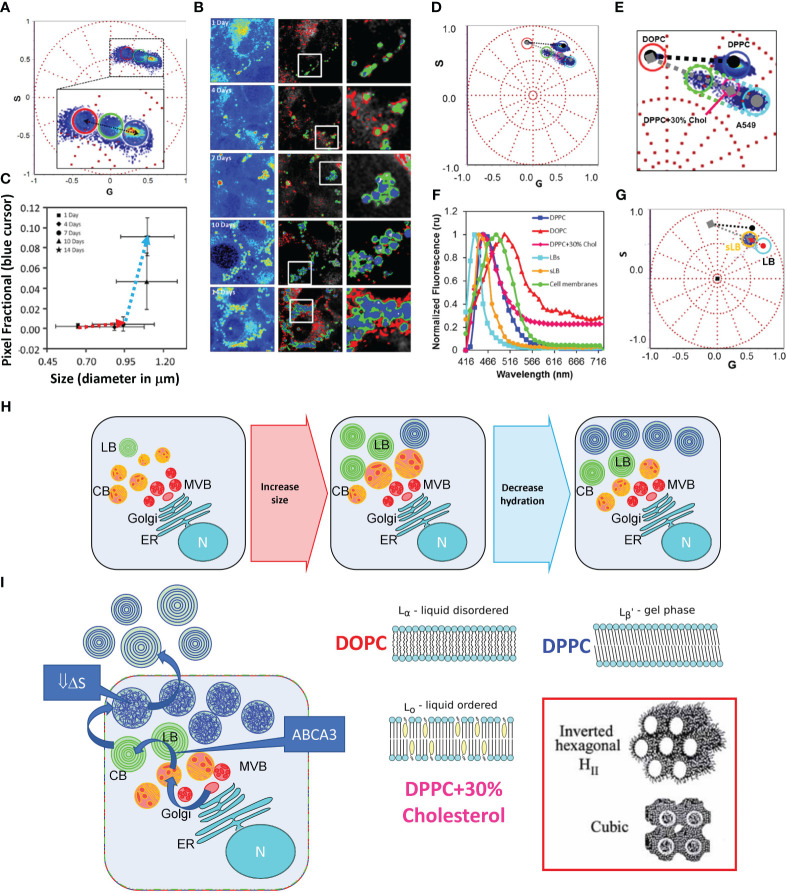
Maturation of Lamellar bodies in A549 cell using HSI and phasor analysis of LAURDAN fluorescence. **(A)** Phasor Plot of the LAURDAN fluorescence spectrum in A549 cells. The cluster includes the different days of post-confluence (1, 4, 7, 10, and 14). LBs were selected using an intensity threshold. The cursors were used to quantify the number of pixels with different spectra or linear combinations of the spectra. **(B)** Representative confocal fluorescent images of A549 cells. The first column contains fluorescence intensity images of LAURDAN in a pseudo-color scale (from blue to red). The second column shows pseudo-colored images of LBs obtained by applying the cursor selection in **(A)** The third column shows a zoom-in of the region of interest in the previous images. **(C)** Plot of LAURDAN fractional pixels intensity (blue cursor in a) versus LB size. Values are represented as mean ± standard deviation. **(D)** Spectral phasor plot of LAURDAN in MLVs displaying different thermodynamic phases. Also, the data for LAURDAN labeled membranes of A549 cells (14 days of post-confluence are included). **(E)** Zoom-in obtained from the figure presented in **(D)** where the liquid order (Lo) – liquid disorder (Ld) (grey dashed line) and solid order (so) – liquid disorder (black dashed line) trajectories are shown. **(F)** LAURDAN emission spectra obtained from intracellular (LBs) and secreted LBs (sLB) plus membranes for A549 cells at 14 days post-confluence. **(G)** Phasor plot analysis of LAURDAN labeled LBs after secretion (sLB, denoted with the orange cursor). Notice that the sLB distribution is inside the trajectory defined by our Ld - Lo references. To highlight the shift, the red arrow shows the shift that occurred upon LB secretion. **(H)** This panel represents a pictorial representation of LB’s maturation process. The sketch illustrates the process supported by the result in panel **(C)**, where LB’s progenitors (Composite Bodies, CB; Multivesicular Bodies, MVB) are growing, but the organelle interior did not change substantially. While, in the second part, the size remains almost invariant, there is significant dehydration due to the organelle filling. **(I)** Cartoon of the thermodynamical hypothesis that explains the results in **(G)** Notice that we hypothesized the occurrence of a significant thermodynamics change during the process of LB’s filling that accumulate energy as a decrease in ΔS. When LBs are secreted to the epithelial lining fluid, the increase in hydration drives the energy to re-organize the membranes. In the right panel are representative images of the membrane models used in panel **(E)** as membrane examples. We hypothesized the occurrence of cubic or hexagonal phases. Figure modified from Malacrida et al. (6).

Once the LB maturation process is complete, they are stored until appropriate signaling induces their secretion. The secreted PS components undergo various transformations, including tubular myelin (TM) formation and adsorption to the air-liquid interface in the alveolus. At this interface, a monolayer is formed, crucial for the tensoactive function of PS, with phospholipids being the essential components (2, 3). In addition to the existence of the monolayer at the interphase, TM and membranous structures (multilayers) have been identified in the hypophase close to the monolayer. These structures are attached to the air-liquid interface and are crucial as a PS reservoir, maintaining and stabilizing the interfacial monolayer. Through electron microscopy, LBs are observed as highly packed bilayer-type structures, essential for their proper functionality. The maturation of the SP-B protein is necessary to achieve a high degree of compactness (5). LBs originate from lysoendosomes with an acidic pH, high calcium concentrations, and proteolytic enzymes (see **Figure 1** sketches). While LBs possess various enzymes and proteins involved in assembling and processing lipids and proteins PS, they lack the enzymes required for lipid synthesis. Surfactant lipids are transported to LBs through vesicular transport (via the Golgi apparatus and multivesicular bodies), non-vesicular transport, and direct diffusion between the endoplasmic reticulum and lamellar bodies. Specific transporter proteins are vital in facilitating these processes (3). Recent studies have identified ATP-binding carrier protein A3 (ABCA3) as a membrane protein responsible for LB biogenesis and surfactant lipid transport through these organelles. This protein utilizes ATP hydrolysis to transport various molecules across cell membranes. Its activity is associated with the accumulation of saturated phospholipid species in surfactant membranes. Additionally, ABCA3 promotes the accumulation of membranes inside multivesicular bodies, which form densely packed LBs upon SP-B protein maturation (7–9). This protein has been considered the key to filling the multivesicular bodies (MVB) to become LB’s. Several other proteins, including lysosomal integral membrane protein-2 (LIMP-2 or SCARB2), Niemann-Pick C1 (NPC1) and Niemann-Pick C2 (NPC2), and P4-type ATPase ATP8A1, have been identified as crucial for LB structure and function. LIMP-2 and NPC1 facilitate cholesterol transport across the lysosomal membrane, with LIMP-2 and NPC1 transporting cholesterol from lysosomes into the cytosol to facilitate cholesterol uptake by the cell (10, 11). However, recent research suggests that LIMP-2’s primary function in LBs may be related to regulating LB phospholipid content rather than cholesterol loading. ATP8A1 is involved in the transmembrane transport of phospholipids, particularly phosphatidylserine (PS) (10–14). An acidic pH within the LBs is crucial for SP-B and SP-C protein processing and lipid packaging. SP-B and SP-C are synthesized as precursors (preprotein) of larger sizes than mature proteins. To become functionally active, they must be proteolytically processed at various stages along the secretion pathway; this process is pH-dependent (15–19). The maintenance of an acidic pH, exocytosis, and surfactant secretion also relies on the presence of functional ion channels. Thus, transmembrane transport of ions and water is necessary. Various isoforms of vacuolar V-ATPase have been identified within LBs, responsible for maintaining an acidic pH by pumping protons into the lamellar bodies. V-ATPase also plays a role in regulating surfactant secretion (14, 20). Pulmonary surfactant secretion occurs through cytosolic calcium-mediated exocytosis of LBs. Stretching of lung tissue during ventilation activates the calcium-dependent secretion pathway in ATII cells by increasing cytosolic calcium levels required to fuse LBs with the ATII cell apical membrane. V-ATPase has been observed to contribute to increased calcium mobilization (20–22). Furthermore, an outwardly directed Na+-K+-2Cl- cotransporter (NKCC1 or SLC12A2), purinergic P2X receptor 4, and vesicular nucleotide transporter (VNUT or SLC17A9) have also been identified (11, 20, 23).

LB hydration plays a critical role in membrane organization. LBs were conceived as concentric multilayer membrane structures that, upon reaching the interface, form multilayers as TM, associate bilayers, and monolayers at the air interphase, resulting in the coexistence of Liquid order/Liquid disorder phases. Thus, the dynamics of LB membranes directly influence their function (24). Studies utilizing electron spin probes and nuclear paramagnetic resonance have demonstrated that LBs exhibit high fluidity at physiological temperatures, influenced by their lipid components (25). Raman spectroscopy studies conducted by Swain and colleagues indicate that lipid content undergoes modifications throughout ATII cell differentiation, with internal lipid content as a distinguishing marker between AT type-I and ATII cells (26). The supramolecular organization of surfactant phospholipids depends on their composition, temperature, chemical strength, PC-saturation, other minor phospholipids, the presence of cholesterol, and the degree of compression at the alveolar interface. Temperature and chemical strength changes can modify thermodynamics, affecting the lipids’ physical properties and degrees of freedom (rotational, diffusional, etc.) (27).

## 2 Role of pulmonary surfactant on lung injury

PS is essential for proper lung function, and its alteration can lead to severe impairment of lung physiology, exacerbating various pathological conditions. Neonatal respiratory distress syndrome (NRDS) is a classic example of PS dysfunction, where deficient secretion of PS by immature ATII cells results in tissue damage characterized by interstitial and alveolar edema, hyaline membrane formation, bronchiolar necrotic lesions, and infiltration of inflammatory cells. The main consequence of NRDS is severe hypoxemia caused by intrapulmonary shunting, abnormal oxygen diffusion, and ventilation-perfusion mismatch (28, 29). Another type of NRDS is meconium aspiration syndrome (MAS), characterized by lung tissue inflammation and endothelial injury. Several alterations of PS phospholipids and proteins have been described in the early phase of MAS because of inflammation. Lysophosphatidylcholine species released by phospholipase A2 increases, whereas the concentration of surfactant proteins B and C increases, affecting the structure and function of PS (30). PS dysfunction also plays a significant role in other respiratory diseases. In adult respiratory distress syndrome (ARDS), characterized by diffuse alveolar damage, PS function can be severely affected. Studies have described significant changes in the composition of PS, including alterations in the phospholipid profile and an increase in the ratio of small to large surfactant aggregates in the bronchoalveolar lavage (BAL) of ARDS patients, contributing to atelectasis formation and decreased lung compliance (31–33). Severe acute respiratory syndrome coronavirus 2 (SARS-CoV-2), responsible for the COVID-19 pandemic, is known to invade ATII cells, impairing the production and adsorption of PS. Furthermore, the composition of PS has been observed to be impaired in COVID-19-associated ARDS, with a decrease in DPPC, the primary phospholipid responsible for surfactant activity (34, 35). The mechanisms underlying PS dysfunction in ARDS are not fully understood. Still, it has been suggested that serum proteins leaking into the alveolar space during lung edema may contribute to PS dysfunction (36). Ventilator-induced lung injury (VILI) is a well-known iatrogenic condition that worsens respiratory function in mechanically ventilated patients. Animal models of VILI have shown that short periods of mechanical ventilation with high tidal volume and zero positive end-expiratory pressure (PEEP) can alter the biophysical properties of PS, leading to increased surface tension and decreased lung compliance (37, 38). The relationship between PS alterations and lung impairment in mechanical ventilation is still debated. Some researchers propose that PS dysfunction induced by mechanical ventilation is a primary factor contributing to VILI and ARDS and that low tidal volume ventilation strategies provide benefits by reducing tissue stretch and preserving PS function. Indeed, a ventilator strategy based on high frequencies and low and controlled tidal volumes (high-frequency oscillatory ventilation) has been proposed to preserve surfactants and decrease the risk of VILI (39). It has also been observed that constant volume tidal ventilation can lead to PS dysfunction, atelectrauma, and VILI, as surfactant inactivation and depletion depend on ventilatory excursions and lung volume (40). Supporting the hypothesis that PS changes occur before the development of VILI, studies in rodent models have shown that treatment with exogenous PS or increasing endogenous pools can restore lung dysfunction in VILI (41, 42). Moreover, Milos et al. studied LBs by transmission electron microscopy in a murine model of VILI and found a decrease in number and impaired function but did not address the mechanism of the alteration (43). High-dose oxygen therapy can induce hyperoxic acute lung injury (HALI), worsening the pulmonary damage caused by mechanical ventilation. Hyperoxia has been shown to affect PS *in vitro*, reducing its surface tension activity. *In vivo* experiments have observed hyperoxia impairs surfactant function and induces oxidative changes in some lipid components. Additionally, exogenous surfactant administration has been found to mitigate lung injury in animal models of hyperoxia (44–51). Furthermore, anesthetic gases themselves can also affect surfactant properties and function. Studies have shown that short-term exposure to sevoflurane can induce changes in the phospholipid composition of PS, leading to impairment of pulmonary mechanics and tissue damage in adult rats (52). PS dysfunction can also occur in respiratory infections. Bacterial components, such as lipopolysaccharide (LPS) found in gram-negative bacteria, can alter the composition of PS and induce surfactant dysfunction *in vitro*. Moreover, alterations in phospholipid profiles like those seen in ARDS have been observed in BAL samples from patients with bacterial pneumonia (32, 53, 54). Other microorganisms, such as Pneumocystis carinii and respiratory syncytial virus (RSV), have also been shown to affect PS function. In a mouse model of Pneumocystis carinii pneumonia (PCP), reduced surface tension activity of PS was observed after infection. In contrast, RSV infection in infants led to a decrease in SP protein concentration (55, 56). Since PS components have antimicrobial properties, their alteration by an infection can contribute to microbial propagation and respiratory failure, with severe consequences for the patient. PS dysfunction also plays a significant role in chronic lung diseases, such as chronic obstructive pulmonary disease (COPD), idiopathic pulmonary fibrosis (IPF), and cigarette smoking, which have been extensively reviewed recently (4). Alterations in the composition and function of PS are key pathogenic factors in the progression of these chronic lung diseases.

As described above, increasing evidence supports that PS is affected by different pathological processes contributing to PS dysfunction and enhancing respiratory disease. Although the alteration of PS composition and the impairment of its function are consistently reported on lung disease, little is known about the mechanism of those changes. Since the LBs are the intracellular form of PS, it is interesting to study biosynthesis and trafficking processes affected by different pathological scenarios. Therefore, studying the intricate mechanisms of PS biology and its trafficking processes, from synthesis to transport to the alveolar surface and reabsorption, is essential to enhance our understanding of its role in lung diseases. In this context, research on bioimaging, obtaining images in a live animal without the need for tissue extraction combination (intravital microscopy) combined with spectroscopy techniques, can provide novel tools to elucidate pathogenic mechanisms and identify potential therapeutic targets.

## 3 Novel bioimaging opportunities to study the intracellular pulmonary surfactant trafficking

The advent of new techniques, such as multiphoton microscopy or its combination with spectroscopic tools such as fluorescence lifetime imaging microscopy (FLIM) or hyperspectral imaging (HSI), enables us to address *in vivo* questions that were inaccessible before (57, 58). Our understanding of the LB organization and maturation comes from transmission electron microscopy (TEM) and, more recently, its combination with cryo-Electron Microscopy (59–62). These fantastic techniques enable unprecedented spatial resolution (nanometer range) with the compromise of using fixed samples. On the other hand, fluorescent microscopy combined with spectroscopy can reach a high temporal (μsec to msec) and considerable spatial resolution (~200 nm). Using the unique solvatochromic properties of LAURDAN fluorescence, Cerrada et al. propose that intracellular LB exists in crystalline-like highly ordered structures, with a highly packed and dehydrated state maintained at supra-physiological temperatures (63). To reach this conclusion, the authors use the generalized polarization function (known as GP), which accounts for a normalized radiometric measurement of the spectral shift suffered by LAURDAN due to relaxation at the membrane interphase (64, 65). This approach expresses intrinsic constraints due to the assumption of two states for LAURDAN fluorescence; therefore, there is no chance of obtaining other potential membrane states than fluid or solid states. Using a model-free method, our group approaches the maturation of LBs on live cells using LAURDAN fluorescence, **Figure 1** (6). Noticeably the combination of hyperspectral imaging with the spectral phasor approach shows a convoluted result from Cerrada et al. that expands our understanding of the intracellular organization of LB. The spectral phasor approach used to analyze LAURDAN HSI data assumes no *a priori* model (66). Hence, the position of the data on the spectral phasor proposes a cluster analysis based on the spectroscopy properties of LAURDAN and the molecular environment where it is located (6). The results indicate that the LBs membrane inside the cell maturates over time, first by increasing size (0-7 days) and then by decreasing the interior hydration (7-14 days), **Figures 1A–C**. Moreover, compared with membranes on crystalline-like (gel), fluid, or liquid-order membranes, LBs do not show the same supramolecular organization, as judged by the fingerprints obtained at the phasor plot using model membranes, **Figures 1D, E**. The clue of which kind of membrane supramolecular organization LBs reach maturation comes from analyzing its organization after secretion. The LBs resume as liquid-order membranes indicating that upon secretion, LBs dramatically change organization due to abrupt change in hydration, **Figures 1G, E**. We concluded by proposing the possibility of having no-lamellar membranes, such as hexagonal structures, due to the change in water dynamics (**Figure 1I**). It is interesting to notice that it was impossible to reach this conclusion without using a model-free approach, such as the phasor approach to analyze LAURDAN fluorescence (6).

While the A549 is an interesting model for intracellular studies of LBs it also has some limitation due to the mutation suffered (67). Other models such as primary ATII cell or differentiate iPS cell are promising great future for its research (68–70). Moreover, novel opportunities are arriving through studying lung organoids or intravital imaging combined with multiphoton microscopy (71–79). The use of lung organoids shows a promising area of research to understand the fundamentals of LBs organogenesis. For instance, these 3D cellular models can be combined with fluorescent proteins to trace LBs molecular markers and correlate this information with electron microscopy (EM) (80, 81). Such approaches are known as correlative light-electron microscopy and are revolutionizing cell biology to study organogenesis and interaction between organelles. In brief, fluorescence microscopy allows molecular marker identification, and EM produces high-resolution imaging of cellular structures with nanometer resolution (82).

On the other hand, multiphoton imaging allows us to explore lung cells in their native environment and exploit the combination with spectroscopy tools such as HSI. Intravital imaging using 2-photon microscopy has intrinsic difficulties for lung imaging due to the movement over time; new computer vision approaches enable compensating for the movement and studying the neutrophil dynamics on a breathing lung (83). On the other hand, there is still an area to be explored with novel approaches in which spectral data is accessible using snap-shoot HSI using the phasor approach (84, 85). Its combination with microscopy tools such as light-sheet microscopy could open unprecedented spatial and temporal resolution to study intracellular trafficking while obtaining spectral data.

## Author contributions

MG, LA, and LM wrote the article. LM and AB conceived and revised the final document. All authors contributed to the article and approved the submitted version.
